# Itraconazole perturbs colorectal cancer dormancy through SUFU-mediated WNT inhibition

**DOI:** 10.1080/23723556.2018.1494950

**Published:** 2018-08-07

**Authors:** Semiramis A. Popova, Simon J. A. Buczacki

**Affiliations:** a Cancer Research UK Cambridge Institute, Robinson Way, Cambridge, UK; b Cambridge Colorectal Unit, Department of Surgery, Addenbrooke’s Hospital, Cambridge, UK

**Keywords:** colorectal cancer, itraconazole, hedgehog, wnt, cancer stem cell, dormancy, plasticity, cell cycle, heterogeneity

## Abstract

Cancer cell dormancy is an important source of treatment failure. We studied the molecular characteristics and functional behaviour of dormant colorectal cancer cells finding them to be a differentiated yet plastic population. Organoid drug screening identified itraconazole perturbs dormancy through non-canonical hedgehog signalling effects on the WNT pathway.

Heterogeneity exists at multiple levels in colorectal cancer (CRC) and over the last decade, major progress has been made cataloguing and quantifying inter-tumoral genetic differences.^1^ More recent research effort has also asserted the existence of profound intra-tumoral genetic diversity present throughout the lifetime of a CRC.^2^ Although this molecular heterogeneity is clearly an important factor in treatment failure, cellular functional heterogeneity can also explain the disparity in response to chemotherapy between patients with similar mutational profiles. Functional heterogeneity can be defined as differences in both the identity and phenotype of cancer cells and has been shown to poorly correlate with mutational background.^3^ Studies seeking to understand functional heterogeneity have tended to focus on understanding the nature of highly clonogenic so-called cancer stem cell populations. Efforts to explore cell cycle-defined functional heterogeneity have been limited despite the well-established connection between cell cycle progression and clonogenic status.

Adjuvant CRC therapy relies on s-phase cytotoxics, which target exclusively dividing cells. Therefore, it is of vital clinical importance to effectively identify and target dormant CRC cell populations as potential drivers of disease recurrence. Prevalent prolonged intermission between primary disease treatment and representation with metastases in CRC patients indicates that reactivation of chemotherapy-resistant quiescent cancer cells may indeed be key in disease relapse. Having previously defined the nature of quiescent cells in the normal murine intestinal epithelium we sought to apply insights from normal stem cell biology to aid the identification of dormant CRC cells.^4^ The study of tumour dormancy is technically difficult as there are only a small number of suitable experimental assays that can be used to identify dormant cells. The most common technique used to identify and isolate dormant cells is through label-retention. Label-retaining assays involve initially marking all cells of interest with a visible label. Following labelling, proliferative cells will gradually dilute out the marker with subsequent divisions whereas dormant cells will retain a visible label (label-retaining cells).

To define the molecular landscape of CRC cell dormancy we applied a label-retaining approach, to a panel of cell lines representing the molecular diversity found in CRC, in combination with transcriptomic profiling.^5^ Cells were grown in spheroid culture to permit the simultaneous culture of both clonogenic and differentiated cells thereby mirroring primary CRC cellular heterogeneity. In other cancers dormant cells have commonly been found to possess cancer stem cell features, however we unexpectedly found dormant CRC cells to reside in a differentiated state. Furthermore, upon re-culture in a permissive environment, we found dormant cells readily re-entered the cell cycle and acquired a cancer stem cell identity. These surprising results are consistent with a recent report from Toshiro Sato’s group who lineage-traced LGR5^+^ (leucine-rich repeat-containing G-protein coupled receptor 5) cancer stem cells and KRT20^+^ (keratin 20) differentiated cells in murine CRC xenografts.^6^ In agreement with our findings, Sato and colleagues observed that following elimination of LGR5^+^ cancer stem cells, KRT20^+^ cells de-differentiated and acquired cancer stem cell features. This plasticity of cancer cell identity and functionality demonstrated by ourselves and the Sato group resembles the previously described reversion of partially differentiated intestinal progenitors to a stem cell state upon perturbation of homeostasis.^4,7^

We hypothesised that perturbing dormancy would render the tumour, as a whole, more sensitive to conventional chemotherapy. Therefore, to identify compounds that could alter the dormant state of label-retaining cells we carried out a functional murine organoid drug screen. The most striking phenotype identified from our drug screen was found with the anti-fungal itraconazole. Itraconazole generated profound organoid collapse, almost complete loss of dormant cells (through an initial proliferative burst) followed by global senescence. We validated our murine organoid findings in human CRC cell lines, human primary and metastatic patient-derived organoids, and performed transcriptomic analysis to examine the mechanism of the itraconazole derived phenotype.

Canonical hedgehog signalling in CRC is predominantly paracrine with pathway activation found in the stromal microenvironment alone.^8^ Itraconazole has previously been shown to inhibit canonical hedgehog signalling by binding smoothened (SMO).^9^ Unexpectedly, we found hedgehog pathway inhibition was inconsistently associated with itraconazole responsiveness. Conversely, WNT pathway inhibition was uniformly correlated with the itraconazole response and phenotype. We hypothesised that suppressor of fused (SUFU) may link an apparent hedgehog pathway inhibitor with WNT inhibition. Indeed, siRNA knockdown of *SUFU* rescued the itraconazole phenotype and WNT signalling thereby confirming the novel effect of itraconazole on the key regulatory pathway of CRC development (Figure 1). Finally, we carried out a series of *in vivo* pre-clinical validation studies indicating that itraconazole causes irreversible tumour growth arrest, WNT pathway inhibition and synergistic activity with classical s-phase cytotoxics.

**Figure 1.**
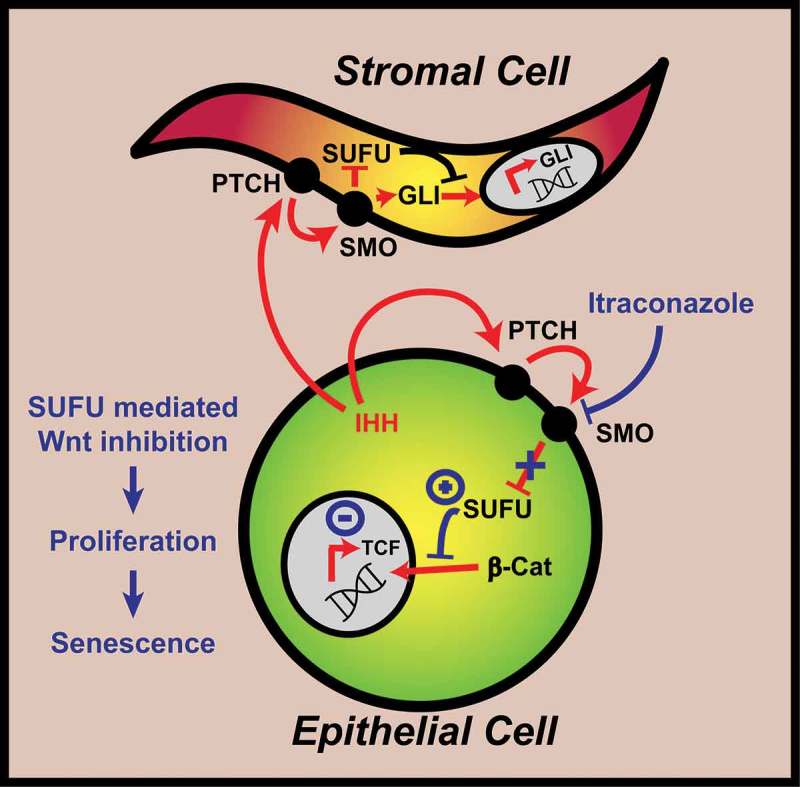
Schematic of the mechanism of itraconazole on colorectal cancer cells. Canonical hedgehog signalling occurs as a paracrine phenomenon in stromal cells resulting in the expression of the glioma-associated oncogene (GLI) family of transcription factors. Itraconazole, inhibits smoothened (SMO) which releases the subsequent inhibition on suppressor of fused (SUFU). Itraconzaole derived SUFU activation in WNT^High^ epithelial tumour cells prevents the nuclear localisation of beta-catenin causing WNT inhibition (diminished *TCF* expression) and a phenotype of proliferation and then global tumour senescence in both dividing and dormant cancer cells.

Autocrine non-canonical hedgehog effects on WNT signalling and differentiation status in CRC has been recently described by Martin Lange’s group.^10^ Our findings and that of Lange and colleagues are highly compatible with the exception that Regan et al. propose that the non-canonical hedgehog derived effect is Patched-1 dependent. However, our data show this to be SMO and SUFU mediated. Interestingly, we found that another classical smoothened inhibitor, cyclopamine failed to reproduce the findings found with itraconazole. This suggests that SMO inhibitors may induce distinctive structural changes which may differentially alter the subsequent interaction between SMO and SUFU and potentially explain the difference between Lange’s findings and ours.

Cumulatively, our study provides evidence that a safe FDA-approved compound generates profound growth retardation in WNT^High^ CRC and also renders dormant cells temporarily vulnerable to cytotoxic therapy prior to entering permanent senescence paving the way for future clinical studies.
